# Unilateral hippocampal infarction associated with an attempted suicide: a case report

**DOI:** 10.1186/1752-1947-8-219

**Published:** 2014-06-23

**Authors:** Yasushi Nishiyori, Masaki Nishida, Katsutoshi Shioda, Shiro Suda, Satoshi Kato

**Affiliations:** 1 Department of Psychiatry, Jichi Medical University, 3311-1, Yakushiji, Shimotsuke, Tochigi 329-0498, Japan

**Keywords:** Suicide attempt, Hippocampal infarction, Ischemic stroke, Glyphosate, MRI

## Abstract

**Introduction:**

In our case report we describe the case of a patient who experienced a stroke in her left hippocampus that was found following an attempted suicide via glyphosate overdose. To the best of our knowledge this is the first case report to describe a hippocampal infarction associated with a drug overdose.

**Case presentation:**

A 64-year-old Japanese woman was brought to our emergency department after ingestion of an unknown dose of glyphosate surfactant herbicide in order to attempt suicide. On admission, she was assumed to be presenting with depression or psychiatric illness, however, sudden-onset memory deficit became apparent. The patient manifested delirium, confusion, and severe anxiety. In addition, short-term memory loss was prominent, with the patient forgetting her attempted suicide. Following an array of standard tests and a brain computed tomography scan (which only showed an old infraction), we performed a magnetic resonance imaging scan and neuropsychological evaluations. The brain magnetic resonance image revealed a small high-intensity lesion in the dorsal part of the left hippocampal body, and memory tests demonstrated severe short-term recall deficits. We diagnosed her with a left hippocampal infarction and administered a course of 75mg of clopidogrel. She gradually became less confused over the course of a week, and a follow-up memory test revealed partial improvement in some domains. No abnormalities were found on a follow-up brain scan. However, despite rehabilitation, memory impairments remain.

**Conclusions:**

It is important to note that had the symptom of short-term memory been absent or less severe, she might have been misdiagnosed with depression or another psychiatric illness. Although a computed tomography scan failed to detect hippocampal lesions, a diffusion-weighted magnetic resonance imaging scan clearly revealed a lesion within the left hippocampus. Therefore, in addition to assessments focusing on psychiatric illnesses that might be the root cause of an attempted suicide, organic factors should be considered along with radiological examination and precise memory assessments for diagnosing similar cases.

## Introduction

Isolated unilateral ischemic strokes confined to the hippocampal region are rare. However, damage to this area may cause sudden memory loss and prolonged cognitive deterioration. Although numerous causes of hippocampal infarction have been reported, an infarction caused by attempted suicide or drug overdose has rarely been described. Here, we report the case of a patient with a unilateral infarction in the hippocampal region that was discovered following an attempted suicide via overdose with glyphosate surfactant herbicide. To the best of our knowledge, this is the first case report to describe a hippocampal infarction associated with a drug overdose. Furthermore, this case demonstrates the importance of evaluating memory and conducting a radiological examination when diagnosing future patients that present with similar symptoms.

## Case presentation

A 64-year-old Japanese woman was brought to our emergency department after ingesting an unknown dose of glyphosate surfactant herbicide in order to attempt suicide. The woman had no history of psychiatric illness or neurological disease, however, she had a history of hypertension and hyperlipidemia, both of which were being medically controlled.

Initially, she did not present with headache, dizziness, numbness, or any other neurological symptoms. She was also able to acknowledge the date and her hospitalization, and was able to describe her attempted suicide. The patient also described being depressed after losing her job three months prior to the event, and that she was not taking any antidepressants.

Several hours after admission she became agitated and confused, apprehensively questioning where she was and why she was at a hospital. Following the manifestation of these symptoms, she was admitted to our psychiatric department.

The patient was found to be disoriented with regards to time and place, did not understand why she was in the hospital, and denied having attempted suicide. Although we initially thought this might have been due to depression or psychiatric illness, it became apparent that she was not able to remember events that had occurred after hospitalization. The patient was conscious of her memory impairment and disorientation, and became agitated about her condition to the point of delirium. As a result, she needed to be confined to a protection room for several days.

**Figure 1:**
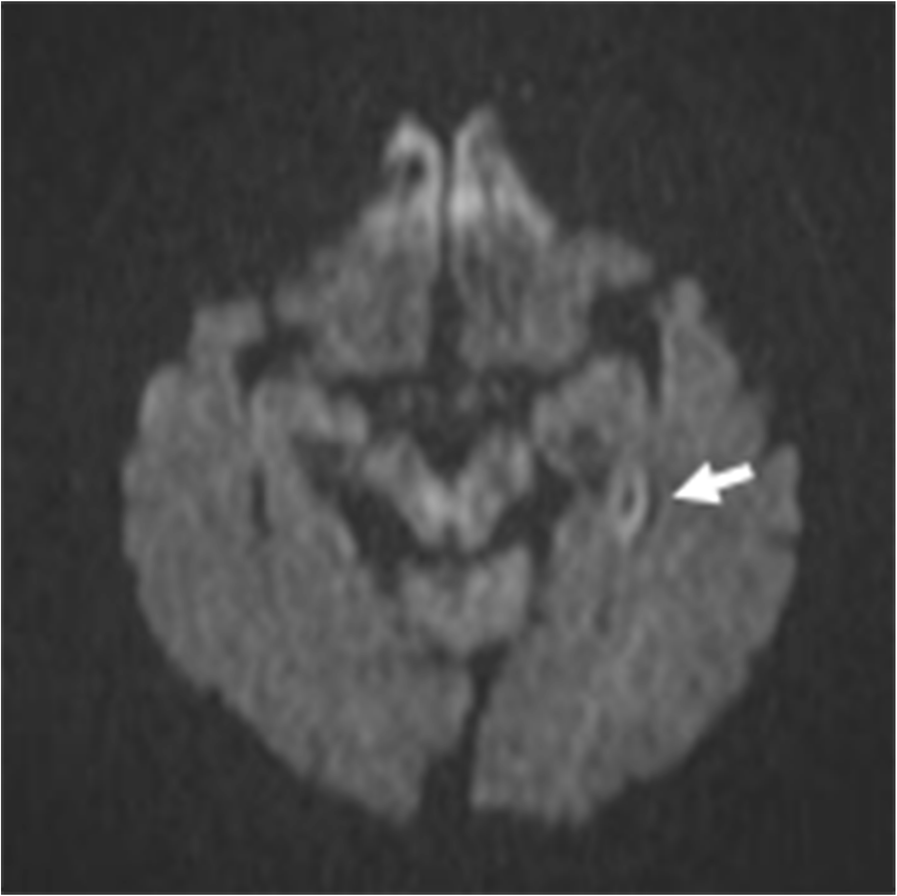
**(Axial) Diffusion-weighted images (DWIs).** DWI magnetic resonance imaging scans showing a high-intensity lesion in the dorsal part of the left hippocampal body (arrow).

**Figure 2:**
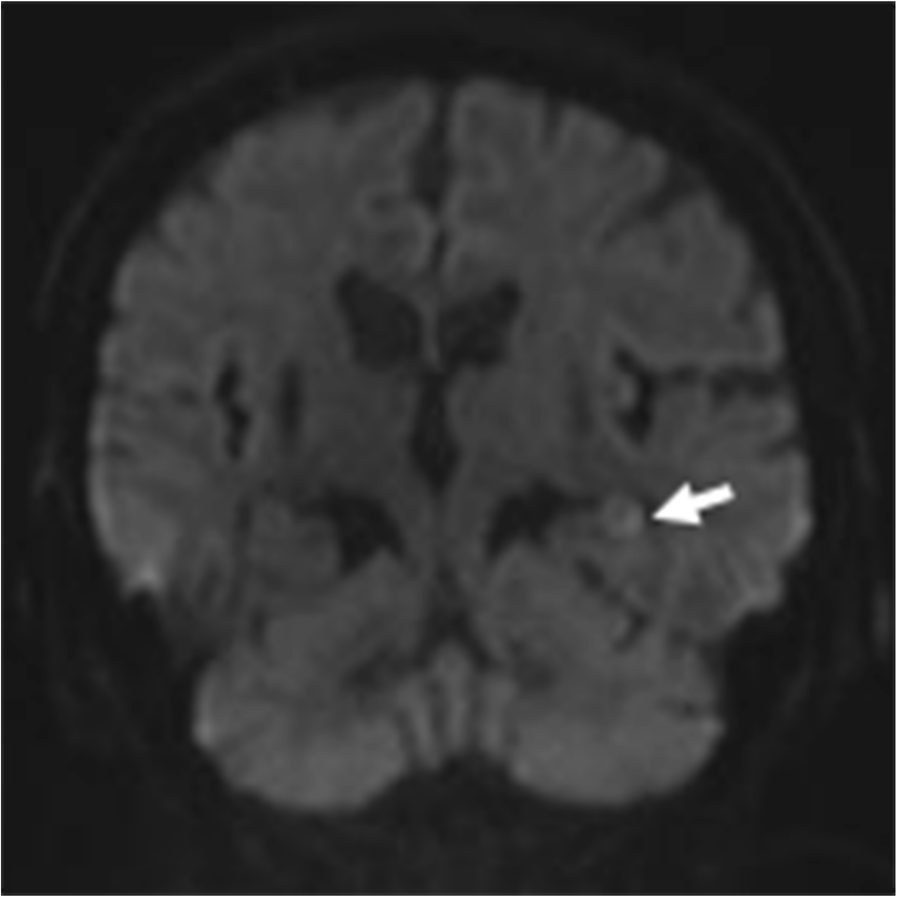
**(Coronal) Diffusion-weighted images (DWIs).** DWI magnetic resonance imaging scans showing a high-intensity lesion in the dorsal part of the left hippocampal body (arrow).

While she was clearly in a state of delirium, a physical examination revealed her to be in a good general condition, with stable vital signs. We observed no focal neuropathological signs except for her memory deficit. Blood tests (including tests for complete blood count, electrolytes, urea, creatinine, and thyroid hormone) and serological test for syphilis yielded normal results. The results of her urine analysis and toxicological screens were also normal. A computed tomography (CT) scan of the brain showed only an old infarction in the bilateral basal ganglia, and we were unable to immediately perform a magnetic resonance imaging (MRI) scan due to her agitated state.A brain MRI scan without contrast was performed on day nine of her hospitalization and revealed a small high-intensity lesion in the dorsal part of the left hippocampal body on the diffusion-weighted image with a corresponding focus of low signal on the apparent diffusion coefficient map. Fluid-attenuated inversion recovery and T2-weighted sequences also revealed a high-intensity signal in a similar area (Figures 1 and 2). An electroencephalogram exhibited regular alpha activity, and the results of her cerebrospinal fluid analysis were within normal limits.

We diagnosed the patient with a left hippocampal infarction and administered a course of 75mg of clopidogrel once daily to be taken orally. Within three weeks since hospitalization, her disorientation to place and state of agitated delirium slightly improved. However, she still could not retain any memory of new events, and her orientation to time was still impaired. We performed the Wechsler adult intelligence scale-third (WAIS-III) and the Wechsler memory scale (WMS-R) for neuropsychological assessment. The WAIS-III revealed an IQ of 70, while the WMS-R yielded the following scores: Verbal memory 52, General memory 64, Delayed memory 65 (Table 1). These results suggest that she had both retrograde and anterograde amnesia.

**Table 1:** Improvement of memory performance during treatment

	**Test at 3 weeks post-hospitalization**	**Test at 2 months post-hospitalization**
WAIS – III*		
Full scale IQ	70	-
Verbal IQ	78	-
Performance IQ	67	-
WMS - R**		
Information and orientation	11	10
Verbal memory	52	74
Visual memory	102	113
General memory	64	84
Attention and concentration	87	102
Delayed recall	65	86

*Wechsler Adult Intelligence Scale - III **Wecheler Memory Scale-Reviced.

A follow-up WMS-R performed two months later revealed a partial improvement in some domains (Table 1), and no abnormalities were found on her follow-up MRI scan. Therefore, she was discharged from the hospital on day 77. Despite a half year of rehabilitation at another hospital, the patient continues to exhibit memory impairments, requiring a notepad to supplement lapses in her ability to recall.

## Discussion

This case was unusual in that a left-side only, hippocampal infarction manifested as memory impairment, clinically similar to a transient global amnesia (TGA). TGA is characterized by the sudden inability to acquire new information (anterograde amnesia) with the preservation of immediate recall and remote memories, but without other neurological signs or symptoms. In patients with TGA, memory deficits typically resolve within 24 hours [1]. However, in this case memory impairment persisted for over five months. Marinkovic *et al.* reported a case of bilateral hippocampal infarction [2] where the clinical course of her condition resembled TGA with the exception of amnesia irreversibility. We suggest that, in patients who display normal head CT scan results and unresolved anterograde amnesia resembling TGA, especially those with significant vascular risk factors, a brain MRI scan should be performed to check for possible hippocampal infarctions.

The CA1 region, which is considered to be involved in TGA-like amnesia [3], has been identified as the hippocampal area that is most vulnerable to ischemia. The CA1 area lies in close proximity to the hippocampal blood supply that branches out from the posterior cerebral artery, and relative hypoperfusion during physical activity or other stress is thought to result in TGA. Various mechanisms for hypoperfusion have been proposed, including increased metabolic demands, increased glutamatergic activity, vasospasm, migraine, and hippocampal oligemia induced by venous congestion [3,4]. Hippocampal infarction might have similar causes. In one report of hippocampal infarction induced by cocaine use [5], the authors suggested that cocaine-induced vasoconstriction was the main cause of cerebral ischemia.

While the cause of hippocampal infarction in this case remains unclear, if we assume that the event occurred before the drug overdose, then this would suggest that a hippocampal infarction could lead to psychiatric symptoms or abnormal behavior (such as attempted suicide). In contrast, if the infarction occurred after the overdose, then this would explain why there was the ‘window period’ in which she was able to acknowledge. In fact, her ability to recall this crucial period suggests that her memory was maintained for at least a few hours after hospitalization. Consequently, this hippocampal infarction was likely to have occurred after this ‘window period’.

Finally, while glyphosate surfactant herbicide poisoning can result in serious multi-organ toxicity including renal failure, hepatic damage, reduced organ perfusion, pulmonary edema, metabolic acidosis, hyperkalemia, ventricular arrhythmia, and shock [6], none of these toxic pathologies were observed in this case. In addition, although she did not exhibit any physical symptoms of herbicide poisoning, it is unclear whether conditions such as depression or psychological stress alone can cause hippocampal infarctions.

In this case, her delirium and agitated state complicated the diagnosis as drug poisoning such as glyphosate surfactant herbicide can be the cause of delirium. Moreover, there is a correlation between the amount of ingested herbicide and the likelihood of serious systemic sequelae [6]. As she did not exhibit any gastrointestinal symptoms, drug poisoning may not have been the main cause of delirium. Meanwhile, several reports suggest that brain lesions supplied by the posterior cerebral artery may cause confusion, presenting with the clinical features of delirium [7]. Moreover, a number of patients have been reported as displaying confusion or agitated delirium associated with hippocampal infarction [8].

## Conclusions

Patients who have attempted suicide are often encountered in hospital emergency rooms. Clinicians tend to attribute the suicides to psychiatric disorders such as depression through initial assessment, missing rarely observed phenomena as described in this case report. Therefore, we hope that reports such as this one may alert clinicians to investigate patients who have attempted suicide as thoroughly as possible. Furthermore, we recommend that an immediate diagnosis should be avoided until all appropriate examinations have been administered, even in cases where symptoms are characteristic of depression.

## Consent

Written informed consent was obtained from the patient for publication of this case report and accompanying images. A copy of the written consent is available for review by the Editor-in-Chief of this journal.

## Abbreviations

CT: Computed tomography; DWI: Diffusion-weighted image; MRI: Magnetic Resonance Imaging; TGA: Transient global amnesia; WAIS-III: Wechsler adult intelligence scale-third; WMS-R: Wechsler memory scale.

## Competing interests

The authors declare that they have no competing interests.

## Authors’ contributions

YN was the principle psychiatrist involved in management of the case. In addition, he was responsible for the review of all literature, medical data collection, and the writing of the manuscript. MN was the psychiatrist on this case, the corresponding author, was involved in management of the patient, was responsible for the literature review associated with the report, was responsible for obtaining patient consent, and also participated in the writing of the manuscript. KS and SS were responsible for assisting with the management of the case. SK was responsible for supervision of management of the patient and was involved in writing the manuscript. All authors have read and approved the final version of this manuscript for publication.
